# Unique immunological profile in patients with COVID-19

**DOI:** 10.1038/s41423-020-00557-9

**Published:** 2020-10-15

**Authors:** Stefania Varchetta, Dalila Mele, Barbara Oliviero, Stefania Mantovani, Serena Ludovisi, Antonella Cerino, Raffaele Bruno, Alberto Castelli, Mario Mosconi, Marco Vecchia, Silvia Roda, Michele Sachs, Catherine Klersy, Mario U. Mondelli

**Affiliations:** 1 Division of Infectious Diseases II and Immunology, Fondazione IRCCS Policlinico San Matteo, Pavia, Italy; 2 Department of Internal Medicine and Therapeutics, University of Pavia, Pavia, Italy; 3 Division of Infectious Diseases I, Fondazione IRCCS Policlinico San Matteo, Pavia, Italy; 4 Department of Clinical, Surgical, Diagnostic, and Pediatric Sciences, University of Pavia, Pavia, Italy; 5 Division of Orthopaedics and Traumatology, Fondazione IRCCS Policlinico San Matteo, Pavia, Italy; 6 Department of Clinical-Surgical, Diagnostic and Pediatric Sciences, University of Pavia, Pavia, Italy; 7 Clinical Epidemiology & Biometry Unit, IRCCS Fondazione Policlinico San Matteo, Pavia, Italy

**Keywords:** COVID-19, NK cells, TIM-3, IL6, Monocytes, Monocytes and macrophages, NK cells, T cells, Viral infection, Interleukins

## Abstract

The relationship between severe acute respiratory syndrome coronavirus-2 (SARS-CoV-2) and host immunity is poorly understood. We performed an extensive analysis of immune responses in 32 patients with severe COVID-19, some of whom succumbed. A control population of healthy subjects was included. Patients with COVID-19 had an altered distribution of peripheral blood lymphocytes, with an increased proportion of mature natural killer (NK) cells and low T-cell numbers. NK cells and CD8^+^ T cells overexpressed T-cell immunoglobulin and mucin domain-3 (TIM-3) and CD69. NK cell exhaustion was attested by increased frequencies of programmed cell death protein 1 (PD-1) positive cells and reduced frequencies of natural killer group 2 member D (NKG2D)-, DNAX accessory molecule-1 (DNAM-1)- and sialic acid-binding Ig-like lectin 7 (Siglec-7)-expressing NK cells, associated with a reduced ability to secrete interferon (IFN)γ. Patients with poor outcome showed a contraction of immature CD56^bright^ and an expansion of mature CD57^+^ FcεRIγ^neg^ adaptive NK cells compared to survivors. Increased serum levels of IL-6 were also more frequently identified in deceased patients compared to survivors. Of note, monocytes secreted abundant quantities of IL-6, IL-8, and IL-1β which persisted at lower levels several weeks after recovery with concomitant normalization of CD69, PD-1 and TIM-3 expression and restoration of CD8^+^ T cell numbers. A hyperactivated/exhausted immune response dominate in severe SARS-CoV-2 infection, probably driven by an uncontrolled secretion of inflammatory cytokines by monocytes. These findings unveil a unique immunological profile in COVID-19 patients that will help to design effective stage-specific treatments for this potentially deadly disease.

## Introduction

Severe Acute Respiratory Syndrome Coronavirus-2 (SARS-CoV-2) is responsible for a pandemic, which has thus far caused over 33 million cases of Coronavirus Disease-19 (COVID-19) with a current case/fatality rate of 3%.^1^ The infection is usually associated with mild symptoms, ranging from low-grade fever to anosmia/dysgeusia, conjunctivitis and diarrhea, but may be responsible for severe interstitial pneumonia, myocarditis, acute kidney injury, acute respiratory distress syndrome (ARDS), multiorgan failure and death.^2^ The systemic involvement of SARS-CoV-2 is thought to derive from the ubiquitous expression of angiotensin converting enzyme 2 receptors in humans, that the virus uses to enter the cells, and which are highly expressed in the epithelial cells of the lungs.^3^ Laboratory tests indicate that patients with severe progression of COVID-19 show signs of secondary haemophagocytic lymphohistiocytosis (HLH), a hyperinflammatory syndrome characterized by a potentially fatal cytokine storm with multiorgan failure, which may be triggered by viral infections.^4^ Akin to HLH, COVID-19 is characterized by lymphopenia, and increased serum ferritin, D-dimer, C-reactive protein (CRP), and lactic-dehydrogenase (LDH), the levels of which are also considered predictors of poor outcome.^5^ Moreover, several serum cytokine concentrations are increased during COVID-19, supporting the hypothesis that virally driven hyperinflammation plays a key pathogenetic role.^2^

Innate immune responses are characterized by a relatively low antiviral type I and type III interferon levels and elevated chemokine expression.^6^ Such inefficient innate antiviral defenses coupled to a high pro-inflammatory response are currently thought to contribute to COVID-19 pathogenesis. This functionally dichotomous innate immune response appears to be at variance with findings in other common respiratory viruses, in which inflammatory chemokine production is balanced by functionally appropriate host innate defenses.^6^ Natural killer (NK) cells have been shown to be reduced in numbers, similarly, but less consistently, to T and B cells particularly in severely ill patients.^7^ Of note, the NKG2A inhibitory receptor frequency^8^ and surface density^9^ were higher in COVID-19 patients compared with healthy controls, and returned to normal after clinical recovery.^8^ Moreover, the immune checkpoint molecule PD-1 was also increased on NK cells.^9^

With respect to adaptive immunity, a consistent finding is the low number of CD4+ and CD8 + T cells, particularly in patients with severe clinical presentations and outcomes.^10–14^ Moreover, T cell exhaustion and skewing toward TH17 has been observed in COVID-19 patients.^12^

Fundamental questions about immune responses to SARS-CoV-2 remain. Beside the immune correlates of protection and duration of immunity, which carry obvious implications for vaccine development, there is a need to clarify unanswered questions on the pathogenesis of COVID-19 and correlates of progression to severe and possibly fatal disease. This prompted us to assess the phenotypic and functional status of NK cells in particular, as well as monocytes and CD4 and CD8 T cells, in patients presenting with clinically moderate to severe interstitial pneumonia emerging in the setting of COVID-19.

## Materials and methods

Thirty-two subjects with radiologically confirmed moderate to severe interstitial pneumonia by chest X-ray and a SARS-CoV-2 RNA positive nasopharyngeal swab or bronchoalveolar lavage constituted the group of patients with COVID-19 (CoV-2). All patients presented with dyspnea requiring variable oxygen supply when they were taken to hospital by ambulance. Blood samples for immunological studies were collected 24–48 h after first examination and before administration of any antiviral and/or immunosuppressive drug. All patients were physically examined in the ER and routine labs and blood gases determined stat. A chest X-ray was performed right after presentation. However, the timing of admission to hospital from onset of symptoms could vary from hours to days, depending on the progression of symptoms and was entirely independent from the investigators. Fifteen CoV-2 patients subsequently succumbed after developing ARDS, and 17 survived. Demographic, clinical, and laboratory findings are reported in Table 1. All patients required oxygen ventilation (Ventimask^®^ - Flexicare Medical, Ltd) delivering from 24 to 60% O_2_ with or without reservoir. Some patients were immediately put on continuous positive air pressure (C-PAP) at presentation. Twenty-five clinically healthy, SARS-CoV-2 RNA negative subjects served as controls (HD, median age 63, range 37–92). Part of this group aged over 75 (median 78.5 range 75–92) was used as control for elderly patients (median 81, range 56–92).

**Table 1:** Clinical characteristics of patients with COVID-19 stratified according to clinical outcome

	Survived *n* = 17	Deceased *n* = 15	Total *n* = 32
Sex (M)	12	12	24
Age, median yrs [range]	63 [32–77]	81 [56–92]	69 [32–92]
Chest X-ray			
- Interstitial pneumonia (IP)	10	6	16
- IP + thickening and/or pleural effusion	7	9	16
Comorbidities^a^			
- No comorbidities	6	2	8
- Presence of one comorbidity	3	6	9
- Presence of two or more	8	7	15
Oxygen support			
- <50%	10	3	13
- >50% or C-PAP	7	12	19
Laboratory data			
Lymphocytes × 10^3^/µl [n.v. 1,5-4,0]	800 [500–2200]	800 [300–1290]	800 [300–2200]
Monocytes c/µl [n.v. 100–1000]	510 [140–1230]	400 [220–480]	500 [140–2100]
PLT × 10^3^/µl [n.v. 150–450]	256 [98–633]	164.5 [94–325]	219.5 [94–633]
CD4 + T cells c/µl [n.v. 410-1590]	280 [144–562]	291 [132–734]	286 [132–734]
CD8 + T cells c/µl [n.v. 190–1140]	189 [32–505]	125 [35–411]	153 [32–505]
NK cells c/µl [n.v. 151–296]	100 [47–378]	95 [6–480]	97.5 [6–480]
B cells c/µl [n.v. 163–288]	80 [29–176]	59 [19–153]	69 [19–176]
ALT U/ml [n.v. 11–34]	36 [11–140]	27 [13–68]	29[11–140]
AST U/ml [n.v.11–39]	37.5 [17–74]	49 [34–137]	39 [17–137]
Length of hospital stay, median days [range]	13 [5–75]	8 [1–50]	12 [1–75]

c/µl = cells/µl, n.v. = normal value, *ALT* Alanine Aminotransferase, *AST* Aspartate Aminotransferase, *PLT* Platelets, *LDH* Lactate Dehydrogenase, *C-RP* C-reactive Protein

^a^Comorbidities: cancer, heart disease, chronic liver disease, hypertension, diabetes mellitus, chronic renal failure

### PBMC isolation and phenotype

PBMC were isolated by standard techniques, resuspended in 90% fetal calf serum (FCS; HyClone, GE Healthcare, South Logan, Utah, USA) + 10% dimethylsulphoxide and stored in liquid nitrogen. Briefly, whole blood was diluted with an equal volume of phosphate-buffered saline (PBS), and diluted blood was layered over Lympholyte (Cedarlane). After centrifugation at 500 × *g* for 30 min at room temperature without the brake applied, the PBMC interface was carefully removed by pipetting and washed with PBS-EDTA by centrifugation at 400 × *g* for 10 min. PBMC pellets were resuspended in PBS containing 2% FCS and washed by centrifugation at 250 × *g* for 10′ at room temperature. Cell numbers were determined by light microscopy count in a Burker chamber. Nonviable cells were identified by staining with trypan blue. Cryopreserved PBMC from CoV-2 subjects and controls were thawed, washed and rested for 30 min in complete medium RPMI-1640 medium supplemented with 10% FCS, 2 mM L-glutamine and antibiotic antimycotic solution (100 U/ml penicillin, 0.1 μg/ml streptomycin, 0.25 μg/ml amphotericin B (Sigma-Aldrich, St. Louis, MO, USA). Subsequently, PBMC were washed and stained for phenotypic analysis using the fluorochrome conjugated antibodies detailed in Supplementary Table 1. For analysis of FcεRIγ expression, cells were treated with Foxp3/Transcription Factor Staining Buffer Set (eBioscience, ThermoFisher Scientific, MA, USA) and subsequently stained with anti-FcεRIγ FITC antibody (Merck Millipore, Burlington, MA, USA) in permeabilization buffer. Data acquisition was performed with FACS Celesta (BD Biosciences).

### NK functional assay

Cell function was evaluated after stimulation for 18 h with 100 U/ml IL-2 (Miltenyi Biotec, Bergisch Gladbach, DE) and 10 ng/ml IL-12 (PeproTech, London, UK). PBMC were then incubated in the presence of MHC class I-non expressing K562 cells (E:T ratio of 5:1), brefeldin A (GolgiPlug) and CD107a (BD Biosciences) for 5 h. The cells were then membrane stained with the following antibodies: anti-CD3, -CD56 (details were listed in Supplementary Table 1). After fixation and permeabilization (Fixation/Permeabilization Solution Kit, BD Biosciences), cells were stained with anti-IFNγ, details are listed in Supplementary Table 1. Analysis was performed with FACS Celesta (BD Biosciences).

### ADCC assay

Antibody-dependent cell-mediated cytotoxicity (ADCC) was performed using SW480 colon cancer cells as targets in the presence or absence of Cetuximab (10 μg/ml). Briefly, unstimulated PBMC rested overnight in complete medium were incubated for 5 h with SW480 colon cancer cells at an E:T ratio of 5:1 in the presence of brefeldin A (GolgiPlug) and CD107a (BD Biosciences). IFNγ production in NK cells was detected by intracellular staining and analyzed by FACS Celesta (BD Biosciences). Gating strategy for NK cell analysis is shown in Supplementary Fig. 2.

### Ex-vivo cytokine detection in monocytes

Within 2 h after sampling, whole-blood was diluted 1:1 with cell culture medium (RPMI 1640) and incubated for 3 h at 37° with brefeldin A. After stimulation, cells were washed and membrane stained with anti-CD14, -CD16 and HLA-DR for 15 min at room temperature. Subsequently, an appropriate amount of BD Pharm Lyse (BD Biosciences) was added to each tube with gentle vortexing and incubated at 37 °C. After this lysis step, blood was washed twice with PBS and cells were fixed and permeabilized (Fixation/Permeabilization Solution Kit, BD Biosciences) at room temperature. Post permeabilization, cells were stained with anti-IL-1β, anti-IL-6, anti-IL-8 at room temperature in the dark for 30 min.

### Cytokine detection

Serum concentrations of soluble IL-1β, −12p70, -6, -8, -10, and TNFα were determined using the BD Cytometric Bead Array (CBA) Human Inflammatory Cytokine kit.

### Statistical analysis

The primary endpoint of the study was to compare immunological features between patients with severe Covid-19 infection and controls. With 30 patients per group, we are able to show a difference of one standard deviation with a power of 90% and 5% type I error. As a secondary endpoint we compared in-hospital mortality between these groups.

Statistical analysis and graphical presentations were performed using GraphPad Software 8.01 (GraphPad Software Inc, La Jolla, CA). Statistical differences between groups were assessed by the non-parametric Mann–Whitney U test. Paired data were analyzed by Wilcoxon signed rank test. Pearson’s correlation was used for the evaluation of bivariate associations.

### Study approval

The study protocol conformed to the ethical guidelines of the 1975 Declaration of Helsinki and was approved by the Institutional Review Board and Ethical Committee of Fondazione IRCCS Policlinico San Matteo (P-20200038894).

## Results

### NK cells show an exhausted phenotype in patients with COVID-19

**Fig. 1:**
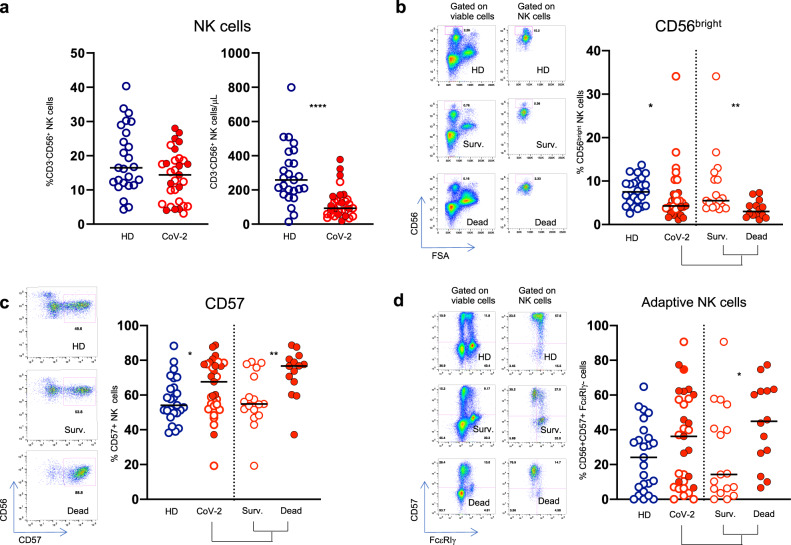
NK cell characterization in SARS-CoV-2 infection. **a** Frequency and absolute count of CD3-CD56 + NK cells. **b** Frequency of CD56^bright^ NK cells, (**c**) Mature CD57^+^ NK cells, (**d**) FcεRIγ negative CD56^+^/CD57^+^ NK cells in PBMC from healthy donors and SARS-CoV-2 patients. Representative dot plots are shown on the left of each graph. Full red symbols indicate deceased patients. Middle bars represent medians. The Mann–Whitney U test was used to compare the two groups. *p < 0.05, **p < 0.01, ***p < 0.001, ****p < 0.0001

Absolute NK cell count was significantly lower in COVID-19 patients compared with controls, whereas the NK cell frequency did not differ between the two groups (Fig. 1a).

**Fig. 2:**
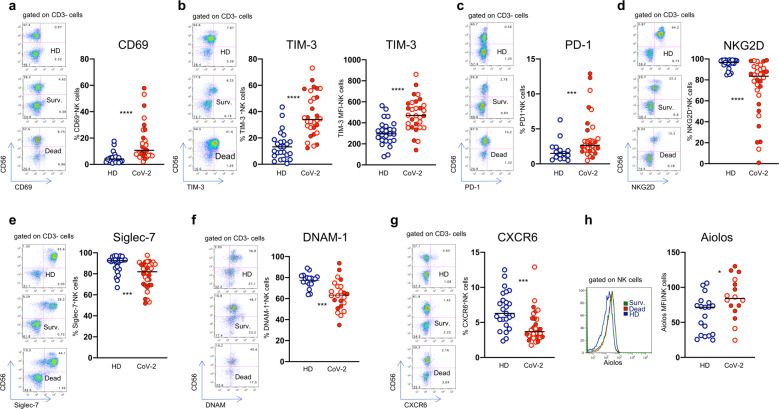
NK cells show an exhausted phenotype in patients with COVID-19. Frequencies of (**a**) CD69- and (**b**) TIM-3-expressing NK cells in healthy donors (HD) and patients with COVID-19 (CoV-2) and corresponding representative dot plots. **c** Levels of expressions of TIM-3 checkpoint molecule on NK cells. Proportion of (**d**) PD-1-, (**e**) NKG2D-, (**f**) Siglec-7-, (**g**) DNAM-1-, and (**h**) CXCR6- positive NK cells and corresponding representative dot plots. **i** Intracellular Aiolos expression in NK cells, expressed as Mean Fluorescence Intensity (MFI). Representative histogram of Aiolos expression on NK cells from a HD (blu line), Survived (green line) and Dead patients (red line). Middle bars represent medians. Full red symbols indicate deceased patients. The Mann–Whitney U test was used to compare the two groups. *p < 0.05, ***p < 0.001, ****p < 0.0001

Analysis of NK cell phenotype showed a reduction of immature CD56^bright^ NK cells (Fig. 1b), and a parallel enrichment in the mature (CD56^dim^/CD57^+^) subset (Fig. 1c) in patients with COVID-19 compared with healthy controls. Of note, these differences were particularly evident in patients who had a fatal outcome (Fig. 1b, c). Moreover, there was a statistically significant increase in adaptive/memory NK cell frequencies in patients who succumbed (Fig. 1d). Since deceased patients were older than survivors (Table 1), we analyzed NK cell phenotype in a small group of elderly healthy controls and no differences were observed between the two control groups (Supplementary Fig. 1a-c). Importantly, there were statistically significant increments in NK cells expressing the CD69 C-type lectin (Fig. 2a), a marker of NK cell activation, and of NK cells expressing the checkpoint molecules TIM-3 (Fig. 2b) and PD-1 (Fig. 2c), whereas the frequencies of NKG2D-, Siglec-7-, DNAM-1-, and CXCR6-expressing NK cells were significantly reduced in COVID-19 patients (Fig. 2d–g). There was an increase of NK cells expressing the transcription factor Aiolos, a member of the Ikaros family that plays an important role in hematopoietic development^15^ (Fig. 2h), while no changes were noted in the frequencies of NKG2C-, NKG2A-, NKp46-, NKp30-, CD16-, TRAIL-expressing NK cells (data not shown).

### NK cells are functionally defective in patients with COVID-19

**Fig. 3:**
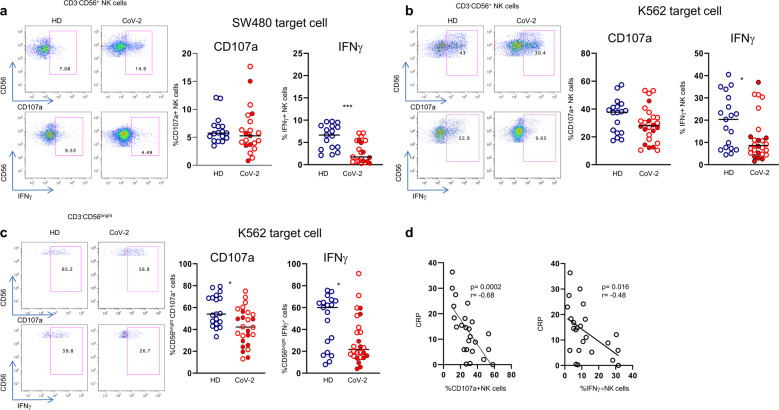
NK cell defective function in patients with COVID-19. **a** Degranulation and IFNγ production by total NK cell after stimulation with SW480. **b**, **c** Degranulation and IFNγ production by total NK cell (CD3^-^/CD56^+^) and CD56^bright^ after stimulation with K562 cells. **a**, **b**, **c** Representative IFNγ and CD107a dot plots in COVID-19 patients (CoV-2) and controls (HD) are shown on the left of each panel. Middle bars represent medians. Full red symbols indicate deceased patients. The Mann–Whitney U test was used to compare the two groups. *p < 0.05. **d** CD107a and IFNγ expression negatively correlate with C-reactive protein (CRP) values. The Pearson test was used to examine correlations

Degranulation activity, IFNγ, and TNFα production were assessed using K562 as target cells, or in ADCC assay using SW480 cells as targets in the presence of cetuximab. With respect to the latter, no changes were observed compared with controls, neither in total NK nor in the adaptive NK cell subset (Supplementary Fig. 2b-c). However, in the abscence of cetuximab, a significant reduction of IFNγ secretion was observed in NK cells from COVID-19 patients compared with healthy donors (Fig. 3a). Importantly, functional experiments with K562 cells showed that NK-cell IFNγ production was significantly reduced in COVID-19 patients compared with healthy donors (Fig. 3b), which was predominantly observed in the CD56^bright^ population associated with a significant reduction in degranulation activity (Fig. 3c). Statistically significant negative correlations were found between the frequency of degranulating (CD107a^+^) and IFNγ-producing NK cells and serum CRP values (Fig. 3d). The negative correlations between NK cell function and CRP indicate that under conditions of exaggerated systemic inflammation NK cells perform poorly, which goes along with NK cell exhaustion.

### A pan-T cell hyperactivated/exhausted phenotype is characteristic of COVID-19

**Fig. 4:**
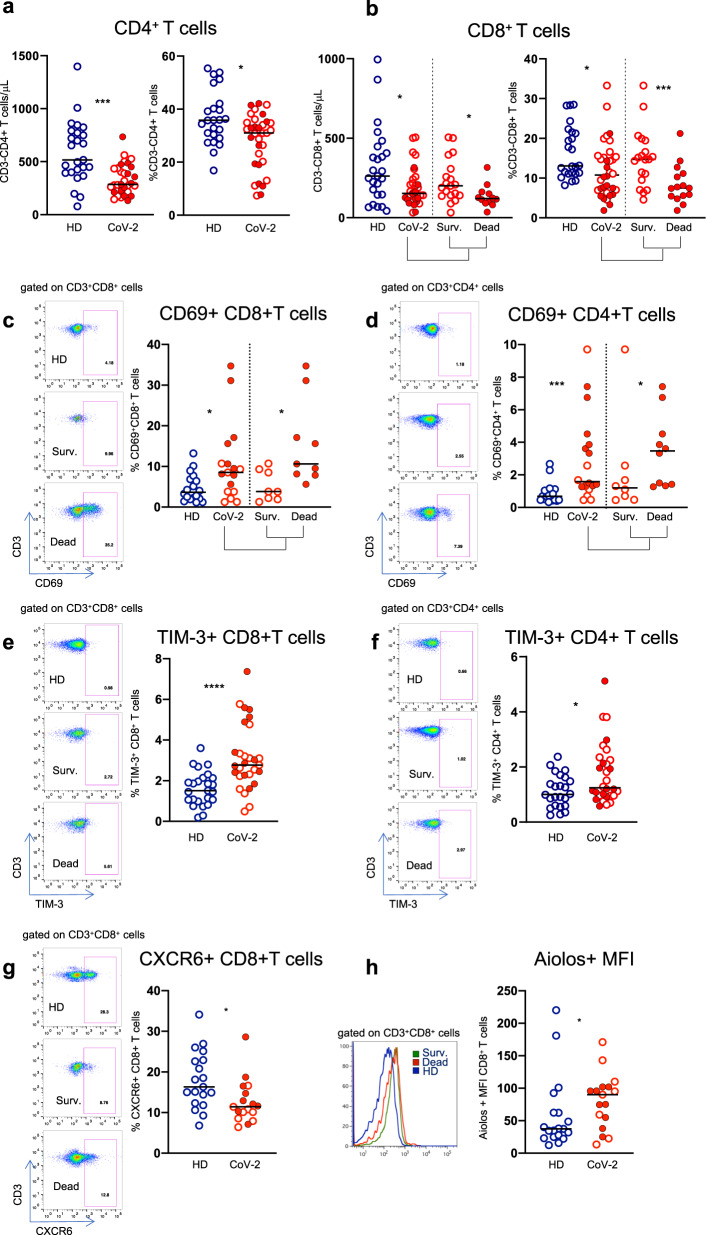
T cells show a hyperactivated/exhausted phenotype during COVID-19 infection. Phenotype of circulating CD4^+^ and CD8^+^ T cells from healthy donor (HD) and SARS-Cov-2 infected patients (CoV-2). Frequencies and absolute numbers of circulating (**a**) CD4 and (**b**) CD8 T cells. **c**, **d** Expression of the CD69 activation marker and (**e**, **f**) of the TIM-3 checkpoint molecule on CD8^+^ and CD4^+^ T cells. **g** CXCR6-expressing CD8 T cells. **e** Intracellular Aiolos expression, expressed as Mean Fluorescence Intensity (MFI). Representative histogram of Aiolos expression on CD8 + T cells from a HD (blu line), Survived (green line) and Dead patients (red line). Middle bars represent medians. Full red symbols indicate deceased patients. The Mann–Whitney U test was used to compare the two groups. *p < 0.05, ***p < 0.001, ****p < 0.0001

Numbers and frequencies of CD4^+^ and CD8^+^ T cells were significantly lower than healthy controls, as frequently reported previously^10–14^ (Fig. 4a, b). Moreover, both CD4^+^ and CD8^+^ T cells from COVID-19 patients overexpressed CD69 (Fig. 4c, d) and TIM-3 (Fig. 4e, f) compared with healthy controls, which is compatible with a hyperactivated pan T-cell exhaustion profile. Of note, direct comparison of CD4^+^ and CD8^+^ T cell phenotypic profile in survivors and deceased patients emphasized the hyperactivated status in both T-cell subsets and a dramatic reduction in the CD8 frequency and T cell count in patients who succumbed compared with those who survived (Fig. 4b). However, CD8 T cell count was not significantly reduced in patients with COVID-19 compared with elderly healthy controls (Supplementary Fig. 1d).

Similarly to NK cells also CD8^+^ T cells expressing CXCR6 were reduced in patients with severe COVID-19 (Fig. 4g), while Aiolos cell density was upregulated (Fig. 4h).

The frequency of CD69-expressing T cells was not different in the two control groups (Supplementary Fig. 1e-f).

### Normal expression of CD69 and TIM-3 is restored on NK and T cells after clinical recovery from COVID-19

**Fig. 5:**
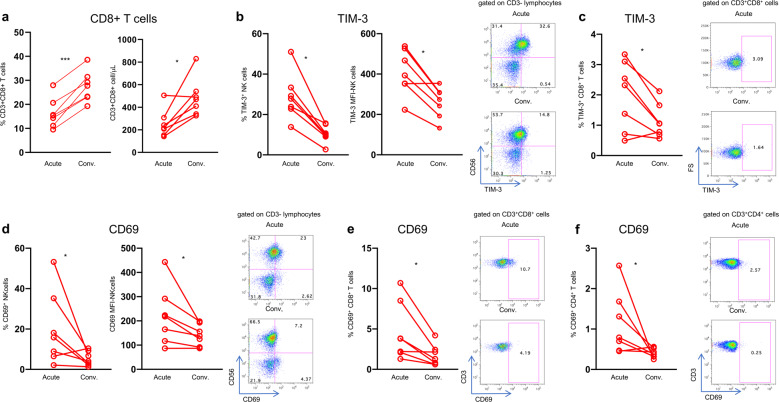
Recovery of CD8 T cells and immune reconstitution in convalescing patients. **a** Frequency and absolute numbers of peripheral blood circulating CD8 + T cells in COVID-19 patients during the acute and convalescent (Conv.) phases. **b** Perecentages (left) and levels of expression (MFI, middle) of TIM-3 molecule by NK cells. Representative dot plots are shown (right). **c** Proportions of TIM-3 positive CD8 + T cells and representative dot plots. **d** Frequency of CD69-expressing NK cells (left) and MFI of the early activation marker CD69 on NK cells (middle). Representative dot plots are shown (right). **e**, **f** Frequency of CD69-expressing CD8 and CD4 T cells and corresponding representative dot plots. Paired data were analyzed by the Wilcoxon signed rank test. *p < 0.05, **p < 0.01, ***p < 0.001

To examine whether the hyperactivated/exhausted phenotype could be restored in patients recovering from COVID-19, we analyzed NK and T cell phenotypes in seven patients during the acute phase and 30–45 days after hospital discharge. All patients experienced an increase in CD8^+^ T cell frequency after recovery (Fig. 5a). Moreover, there was a significant reduction of TIM-3-expressing NK (Fig. 5b) and CD8^+^ T cells (Fig. 5c) and a significant reduction of CD69-expressing cells of all lineages thus far analyzed (Fig. 5d–f). In contrast, NK cell functional recovery was not observed in all patients suggesting that functional changes induced by SARS-CoV-2 were more pervasive than expected (Supplementary Fig. 3a). The proportion of PD-1-expressing NK cells decreased after recovery in 5 of 7 patients, however this did not reach statistical significance (*p* = 0.078, Supplementary Fig. 3b).

### Monocytes release high levels of inflammatory cytokines in sera of patients with COVID-19

**Fig. 6:**
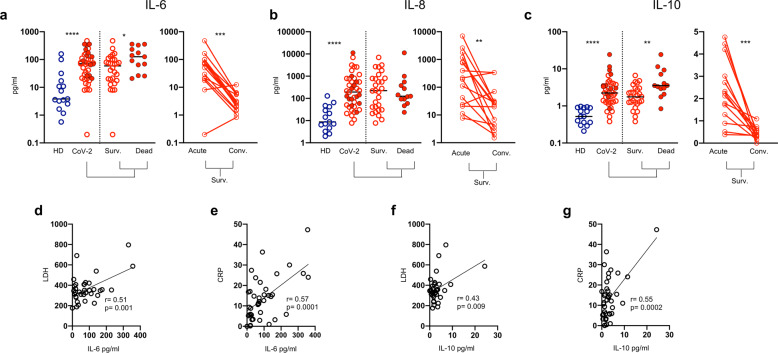
Elevated serum inflammatory cytokines normalize after recovery from SARS-CoV-2 infection. **a**–**c** Left Panel: Serum levels of (**a**) IL-6, (**b**) IL-8 and (**c**) IL-10 in healthy donors (HD) and SARS-CoV-2 patients (CoV-2). Middle bars represent medians. Full red symbols indicate deceased patients. The Mann–Whitney U test was used to compare the two groups. **a**–**c** Right panel: Serum levels of (**a**) IL-6, (**b**) IL-8 and (**c**) IL-10 in the acute phase and after recovery. Paired data were analyzed by the Wilcoxon signed rank test. *p < 0.05, **p < 0.01, ***p < 0.001, ****p < 0.0001. **d**, **e** Correlation of IL-6 with LDH and CRP. **f**, **g** Correlation of IL-10 with LDH and PCR. The Pearson test was used to examine correlations

We examined serum inflammatory cytokines in all patients with COVID-19, including those with favorable and poor outcome. The following cytokines were analyzed: IL-6, IL-8, IL-1β, TNFα, IL-12p70, and IL-10. There were statistically significant increments of serum IL-6, IL-8 and IL-10 levels in patients with COVID-19, consistent with the hyperinflammatory syndrome that is so characteristic of severe and progressive disease, which rapidly dropped during convalescence (Fig. 6a–c). Moreover, serum levels of IL-6 and IL-10 were significantly higher in patients who succumbed compared with those who survived (Fig. 6a, c). Serum IL-6 and IL-10 levels directly correlated with serum CRP and lactic dehydrogenase values (Fig. 6d–g).

**Fig. 7:**
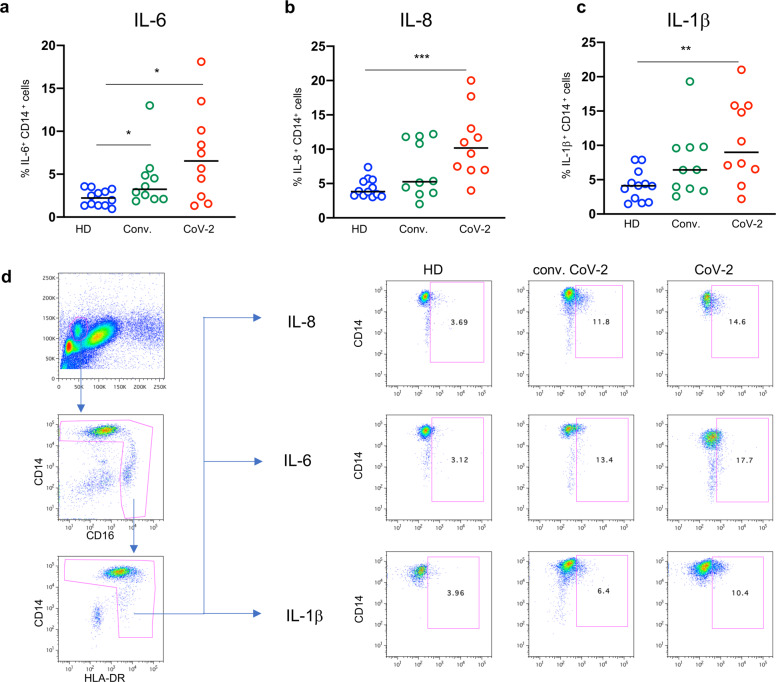
Serum inflammatory cytokines are produced by monocytes in COVID-19. Cytokine detection in ex vivo freshly isolated monocytes from healthy donors (HD), patients with COVID-19 (CoV-2) and recovered individuals (Conv.). **a** IL-6-, (**b**) IL-8- and (**c**) IL-1β-producing monocytes. **a**–**c** Middle bars represent medians. **d** Representative dot plots illustrate the gating strategy used. The Mann–Whitney U test was used to compare the two groups. *p < 0.05, **p < 0.01, ***p < 0.001, ****p < 0.0001

Experiments performed with ex vivo freshly isolated monocytes showed that unstimulated monocytes from patients with COVID-19 were able to spontaneously secrete high levels of IL-6, IL-8 and IL-1β (Fig. 7a–c) which persisted, even though at lower levels, in recovered individuals for several weeks after recovery (Fig. 7a–c). Analysis of classical, intermediate and non-classical monocytes showed that the three populations were all able to secrete increased levels of cytokines in COVID-19 patients (Supplementary Fig. 4). There was a statistically significant correlation between serum IL-1β levels and peripheral blood monocyte count (*r* = 0.42, *p* = 0.007).

## Discussion

There is limited information on PBMC phenotype and function in patients with COVID-19. Here we had the opportunity to evaluate patients admitted to hospital because of largely severe COVID-19 interstitial pneumonia and to compare them with healthy controls. The latter group included a number of healthy elderly individuals, which provided appropriate controls for the group of patients who succumbed because of severe COVID-19, who were significantly older than those who survived. With the exception of CD8 T cell counts, the lack of statistically significant differences between data collected from the whole control group and the elder control group confirmed the robustness of our findings and represent a unique asset to the study, since most immunological studies in this setting lack appropriate controls of age similar to that of the patients.

We have shown here that the global NK and T cell characteristics in COVID-19 infection were compatible with a dysfunctional/exhausted phenotype consequent to hyperactivation, as shown by overexpression of CD69, a marker of activation, and TIM-3 on NK, CD4 and CD8 T cells. TIM-3 is a negative regulator of immune cell function; indeed, engagement with its ligands induces T and NK cell exhaustion in different viral infections^16,17^ and cancer.^18^ Moreover, TIM-3 blockade rescues impaired NK cell function.^19,20^ This generalized overexpression of TIM-3 and CD69, reveals a remarkably consistent and multifaceted hyperactivation and exhaustion profile of the two arms of immunity in this clinical setting. Further ground to support an exhausted phenotype of NK cells comes from overexpression of PD-1, in agreement with a recently published study,^9^ which appears to be more pervasively changed than TIM-3, since it did not completely normalize after recovery.

The reduced frequencies of the activating NK receptor NKG2D and Siglec-7, a diminished expression of which has been associated with reduced NK cell function in viral infections,^21,22^ may also contribute to reduce NK cell effector function in this setting. Interestingly, IL-6, that is present in large excess in sera of patients with COVID-19, may down-regulate NKG2D on NK cells, leading to impairment of NK activity.^23,24^ In keeping with a NK dysfunctional phenotype, IFNγ secretion by NK cells was impaired in COVID-19 patients, particularly in the CD56^bright^ subset. These data are in agreement with another study,^8^ which showed that NK and CD8 T cells are functionally impaired during COVID-19 infection. The hyperinflammatory state is likely responsible for NK and T-cell exhaustion following an exaggerated virus-driven activation, as shown by the persistently high serum concentrations of inflammatory cytokines, namely IL-6, the levels of which are also higher in patients with poor outcome. The enrichment of inflammatory cytokines lends support to the hypothesis that COVID-19 resembles in part to the macrophage-activation syndrome (MAS) which is thought to be closely related to HLH,^25^ an uncommon life-threatening disorder of severe hyperinflammation caused by uncontrolled proliferation of monocytes/macrophages that secrete high levels of inflammatory cytokines. This has been clearly shown in our study and provides mechanistic insights into the source of inflammatory cytokines produced during the course of COVID-19 that persist for several weeks after recovery. Of note, patterns similar to CRS have been described for COVID-19 and SARS.^2,26,27^

The immature CD56^bright^ NK cell population was underrepresented in COVID-19 patients, particularly in those who subsequently died. Moreover, CD57, a marker of highly mature NK cells, was significantly increased in COVID-19 NK cells. CD57 positive NK cells are less responsive to cytokine stimulation but are more responsive to signal through the CD16 receptor. Our findings are in line with a recently published paper,^28^ but at variance with another study showing that the proportion of mature NK cells was markedly lower in patients with ARDS, postulating that these changes would be responsible for the most severe manifestations of COVID-19.^9^ The reasons for such discrepancies are not immediately apparent and may be due to differences in patient characteristics. Interestingly, the Aiolos transcription factor was also increased in NK and CD8 T cells of these patients. Aiolos has been recognized as an important regulator of NK cell maturation and function,^29^ as it is required for maximal IFNγ secretion and for the full control of viral infection. Interestingly, the gene encoding for Aiolos (*IKZF3*), is induced in exhausted T cells^30,31^ indicating a possible association with the observed exhausted T cell phenotype. Interestingly, CXCR6 expression was reduced on NK and CD8 T cells, probably as a consequence of these cells homing to the lung, where the ligand of CXCR6, CXCL16, is highly expressed.^32,33^ Of note, a significant increase of CXCL16 has been described in the serum of COVID-19 patients.^6^

Despite it is still difficult at this early stage to precisely frame COVID-19 within an immunologically coherent clinical entity, several peculiarities have emerged that contribute to the uniqueness of its immune profile, ranging from T-cell and NK-cell exhaustion to patterns compatible with MAS, even though, unlike the latter, IFNγ seems to be normal or suppressed in this setting.^25^ Our data contribute to the characterization of the immunological profile of patients with poor prognosis, who show an enrichment in terminally differentiated, functionally exhausted NK cells, hyperactivated T cells and an increased level of IL-6, with monocytes secreting large amounts of inflammatory cytokines in vitro. Since TIM-3, PD-1, and NKG2A are druggable checkpoint molecules that have been shown to harness NK and T cell immune responses in cancer it may be envisaged to use checkpoint inhibitors to unleash their antiviral activity.^34^ This should be carefully planned and controlled to avoid worsening of the hyperinflammatory state typical of severe COVID-19. Understanding the dynamics and the quality of immune responses to SARS-CoV-2 will provide invaluable translational information to design effective stage-specific treatments for this potentially deadly disease.

## Supplementary information

Supplementary Figure 1

Supplementary Figure 2

Supplementary Figure 3

Supplementary Figure 4

Supplementary Table 1
