# Supplementary material for: Unique immunological profile in patients with COVID-19
Source: Cell Mol Immunol. 2020 Oct 15;18(3):604–12. doi: 10.1038/s41423-020-00557-9 (PMC7557230; doi:10.1038/s41423-020-00557-9)
Supplement: Supplementary file 1 — Supplementary Figure 1 [file 41423_2020_557_MOESM1_ESM.pdf]

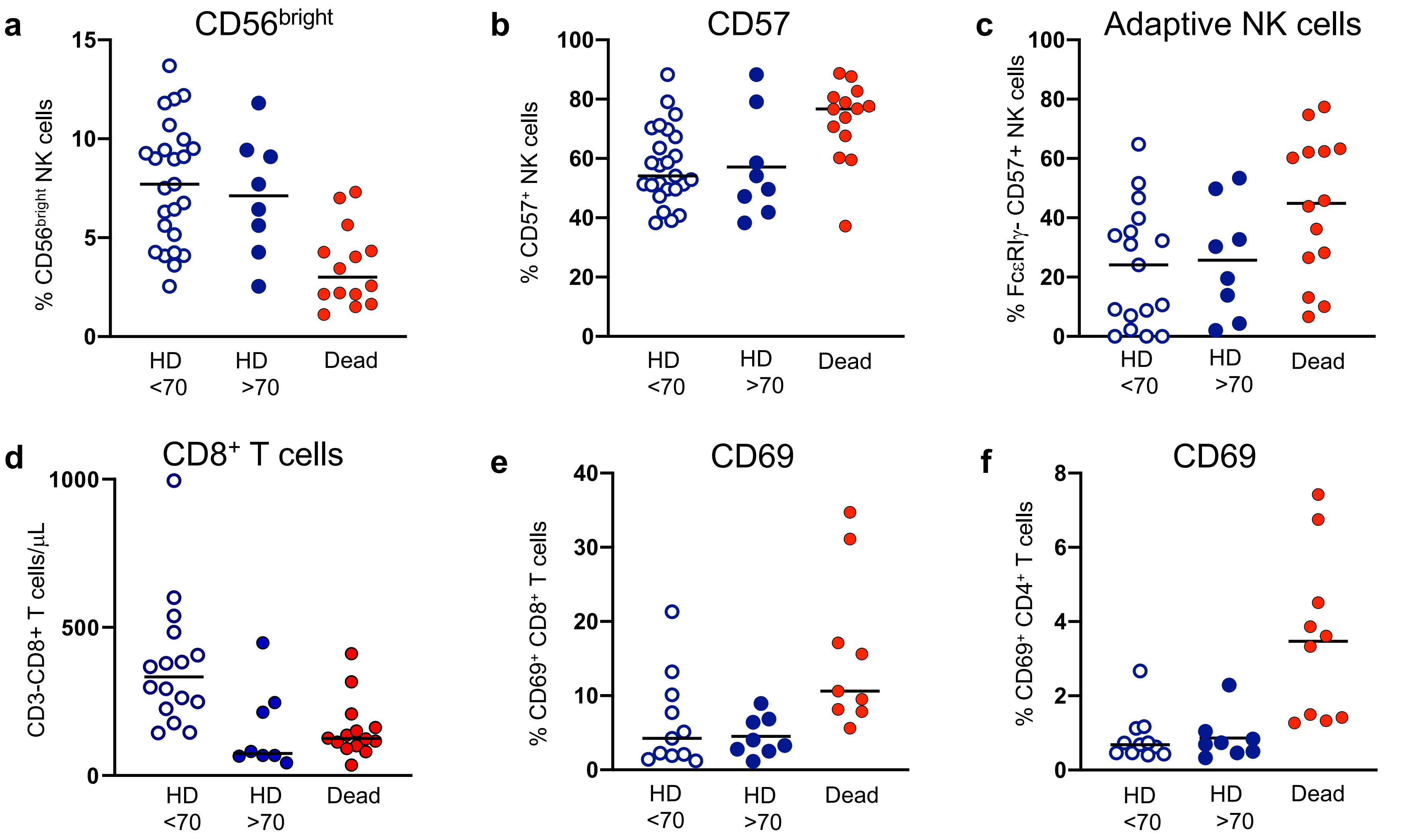

Supplementary Fig. 1. Impact of aging on immune cell phenotype. a-c. Frequencies of CD56^{bright}, mature CD57+ and adaptive NK cells in patients who succumbed (Dead), in age-matched healthy subjects (HD>70) and in the younger control groups (HD<70). **d** Absolute numbers of peripheral blood circulating CD8^{+} T cells and **e, f** Expression of CD69 on CD8^{+} and CD4^{+} T cells in patients who succumbed, in age-matched healthy subjects and in the younger control groups. Middle bars represent median values.
