# Supplementary material for: Unique immunological profile in patients with COVID-19
Source: Cell Mol Immunol. 2020 Oct 15;18(3):604–12. doi: 10.1038/s41423-020-00557-9 (PMC7557230; doi:10.1038/s41423-020-00557-9)
Supplement: Supplementary file 2 — Supplementary Figure 2 [file 41423_2020_557_MOESM2_ESM.pdf]

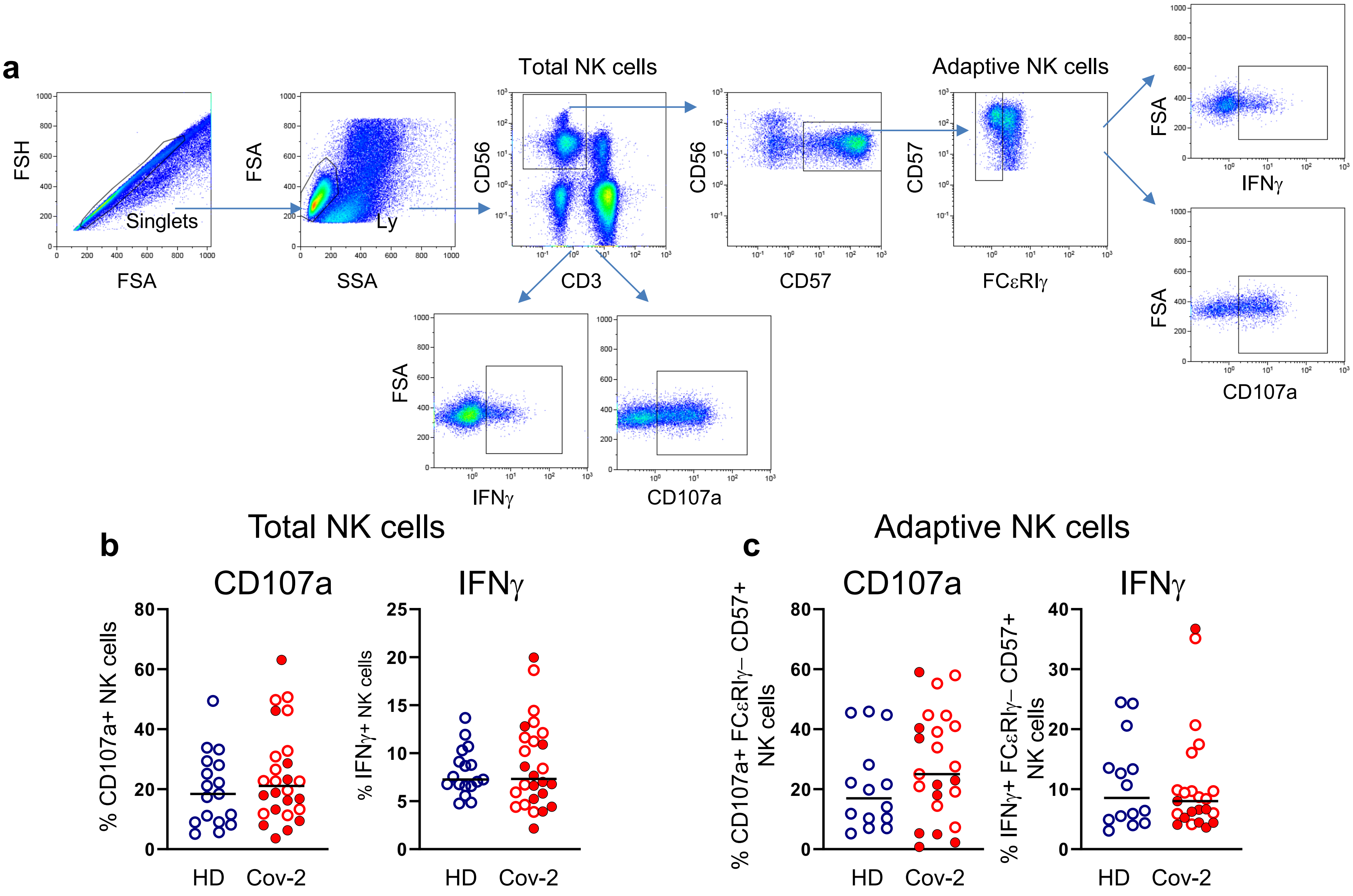

Supplementary Fig. 2. Antibody-dependent cell-mediated cytotoxicity (ADCC) in COVID-19 patients. ADCC was evaluated in Healthy Donors (HD) and in patients with COVID-19 toward SW480 target cells in the presence of Cetuximab. **a** Representative dot plots illustrate the gating strategy used to analyse expression of CD107a and IFNγ in total and adaptive NK cells. **b, c** Expression of CD107a and IFNγ on total and adaptive NK cells. Middle bars represent medians. Full red symbols indicate deceased patients.
