# Supplementary material for: Unique immunological profile in patients with COVID-19
Source: Cell Mol Immunol. 2020 Oct 15;18(3):604–12. doi: 10.1038/s41423-020-00557-9 (PMC7557230; doi:10.1038/s41423-020-00557-9)
Supplement: Supplementary file 3 — Supplementary Figure 3 [file 41423_2020_557_MOESM3_ESM.pdf]

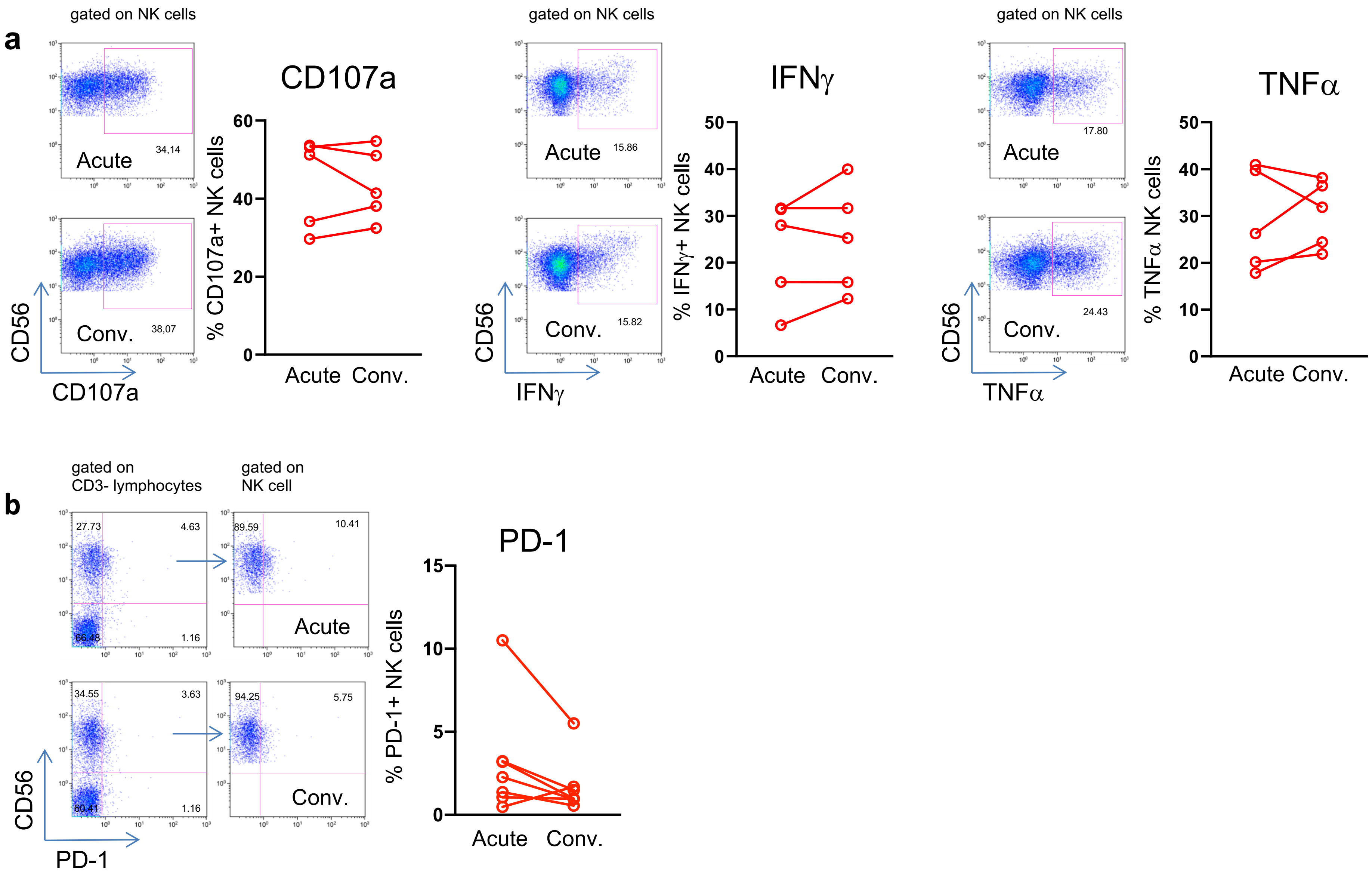

Supplementary Fig. 3. a NK cell degranulation, IFNγ and TNFα production in COVID-19 patients during acute disease and convalescence**. b** frequency of PD-1 expressing NK cells.during acute disease and convalescence.
