# Supplementary material for: Unique immunological profile in patients with COVID-19
Source: Cell Mol Immunol. 2020 Oct 15;18(3):604–12. doi: 10.1038/s41423-020-00557-9 (PMC7557230; doi:10.1038/s41423-020-00557-9)
Supplement: Supplementary file 4 — Supplementary Figure 4 [file 41423_2020_557_MOESM4_ESM.pdf]

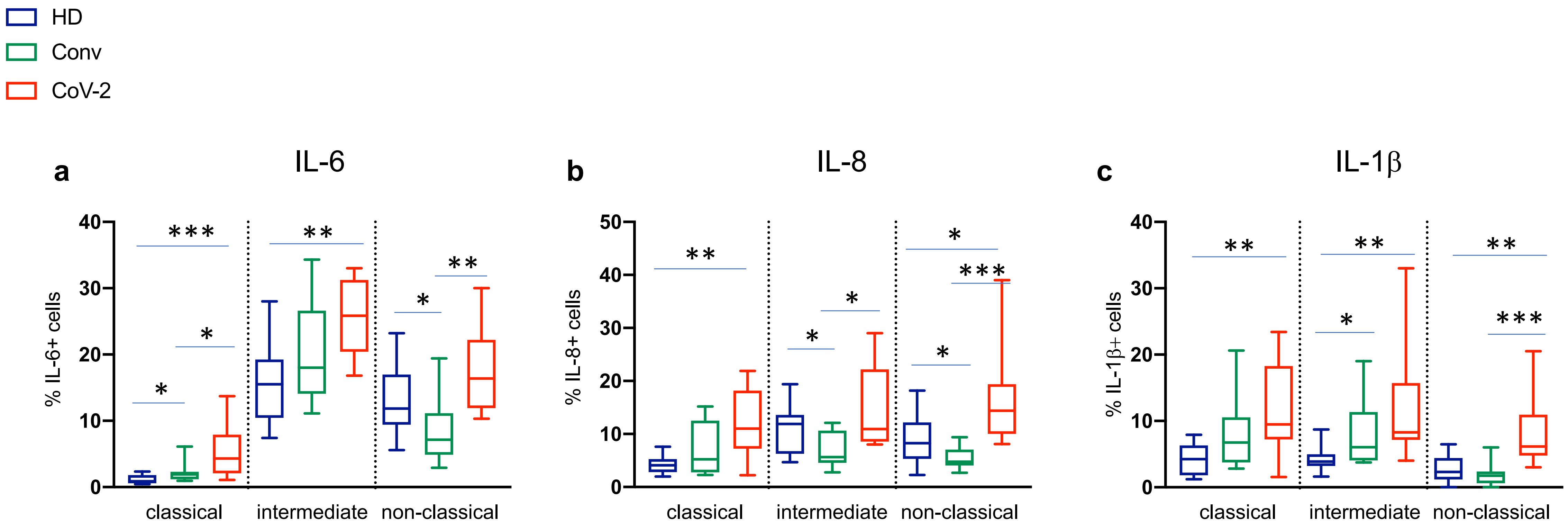

Supplementary Fig. 4. Cytokine production in classical, intermediate and non-classical monocytes. Frequencies of **a** IL-6-, **b** IL-8- and **c** IL-1β secreting monocyte populations in healthy subjects (HD), Covid convalescent subjects (Conv.) and in hospitalized Covid patients (CoV-2).
