# Supplementary material for: Unique immunological profile in patients with COVID-19
Source: Cell Mol Immunol. 2020 Oct 15;18(3):604–12. doi: 10.1038/s41423-020-00557-9 (PMC7557230; doi:10.1038/s41423-020-00557-9)
Supplement: Supplementary file 5 — Supplementary Table 1 [file 41423_2020_557_MOESM5_ESM.pdf]

Supplementary Table 1. Monoclonal antibodies used for flow cytometry.

**Name**	**Supplier**	**Clone**
Anti-human CD3 APC-Cy7	BD Biosciences San Diego, CA, USA	SK7
Anti-human CD3 BV421	BD Biosciences San Diego, CA, USA	UCHT-1
Anti-human CD3-BV510	BD Biosciences San Diego, CA, USA	UCHT-1
Anti-human CD4 BB700	BD Biosciences San Diego, CA, USA	RPA-T4
Anti-human CD8 BV510	BD Biosciences San Diego, CA, USA	SK1
Anti-human CD14 BV605	BD Biosciences San Diego, CA, USA	M5E2
Anti-human CD16 BV786	BD Biosciences San Diego, CA, USA	3G8
Anti-human CD56BB700	BD Biosciences San Diego, CA, USA	NCAM16.2
Anti-human CD57 BV421	BD Biosciences San Diego, CA, USA	NK-1
Anti-human CD69 APC	BD Biosciences San Diego, CA, USA	FN50
Anti-human CD107a BV786	BD Biosciences San Diego, CA, USA	H4A3
Anti-human CD226 BB515	BD Biosciences San Diego, CA, USA	DX11
Anti-human CD253 PE	BioLegend San Diego, CA, USA	RIK-2
Anti-human CD328 APC	BioLegend San Diego, CA, USA	6-434
Anti-human AIOLOS	BD Biosciences San Diego, CA, USA	S50-895
Anti-human CXCR6 BV421	BD Biosciences San Diego, CA, USA	13B1E5
Anti-human HLA-DR BV605	BD Biosciences San Diego, CA, USA	G46-6
Anti-human IFN-γ APC	BD Biosciences San Diego, CA, USA	B27
Anti-human IFN-γ PE	BD Biosciences San Diego, CA, USA	B27
Anti-human IL1β PE	BD Biosciences San Diego, CA, USA	A510
Anti-human IL6 BV421	BD Biosciences San Diego, CA, USA	MQ2-13A5
Anti-human IL8 BV510	BD Biosciences San Diego, CA, USA	G264-8

Anti-human NKp30 BV786	BD Biosciences San Diego, CA, USA	P30-15
Anti-human NKp46 BV421	BD Biosciences San Diego, CA, USA	9E2
Anti-human NKG2A APC	BeckmanCoulter, Fullerton, CA	Z199
Anti-human NKG2C PE	R&D System, Minneapolis, MIN, USA	134591
Anti-human NKG2D PE-CF594	BD Biosciences San Diego, CA, USA	1D11
Anti-human PD1 BV605	BD Biosciences San Diego, CA, USA	EH12
Anti-human Tim-3 BB515	BD Biosciences San Diego, CA, USA	7D3
Anti-human TNFα APC	BD Biosciences San Diego, CA, USA	MAb11
Anti-human FcεRIγ FITC	Merck Millipore, Burlington, MA, USA	Rabbit polyclonal IgG
